# Genetic Diversification in a New Guinean Frog Genus (*Mantophryne*, Microhylidae) was Driven by Ancient Tectonic Activity and Climate Reorganisation

**DOI:** 10.1002/ece3.73291

**Published:** 2026-05-06

**Authors:** Rebecca S. Morris, Fred Kraus, V. Deepak, Saunak Pal, Stephen J. Richards, Simon T. Maddock

**Affiliations:** ^1^ School of Natural and Environmental Sciences Newcastle University Newcastle UK; ^2^ School of Science, Engineering & Environment University of Salford Salford UK; ^3^ Leverhulme Centre for Anthropocene Biodiversity University of York York UK; ^4^ Department of Ecology and Evolutionary Biology University of Michigan Ann Arbor Michigan USA; ^5^ Natural History Museum London UK; ^6^ Herpetology Department South Australian Museum Adelaide Australia; ^7^ Island Biodiversity and Conservation Centre University of Seychelles Anse Royale Seychelles

**Keywords:** amphibians, biogeography, evolution, genetics, Western Melanesia

## Abstract

Western Melanesia sits at the junction of three major tectonic plates, characterised by dynamic plate interactions and geological diversity. Tectonic activity in this region has given rise to modern New Guinea, a relatively young island with extreme topographic heterogeneity and evolutionarily complex biota. New Guinea—particularly the East Papuan Composite Terrane (EPCT)—supports exceptional frog diversity; however, evolutionary histories of many frogs of the EPCT are poorly understood. The microhylid frog genus *Mantophryne* currently includes five described species (*M. axanthogaster*, *M. insignis*, *M. louisiadensis*, *M. menziesi* and *M. lateralis*), with the first four confined to small portions of the EPCT and the last distributed across the eastern half of New Guinea. The biogeographic history of these frogs remained incompletely resolved at the time of our study. We estimate that *Mantophryne* diverged from other microhylid genera approximately 14.4 million years ago (MYA) and began to speciate ca. 8.5 MYA. We recover eight distinct lineages within frogs assigned to *M. lateralis* , which started to diverge ca. 5.5 MYA. Dispersal of *Mantophryne* out of the EPCT occurred in the late Pliocene, around the time the Central Highlands expanded eastward to connect with the EPCT. Six pairs of sister lineages arose during a time of significant climate reorganisation in the Late Pliocene—Early Pleistocene when it is possible that areas to the north of the Central Highlands experienced increased precipitation, while areas south of the Central Highlands experienced lower rainfall, resulting in contraction of rainforests and expansion of savannahs. We did not identify any significant differences in habitus between lineages, other than *M. louisiadensis* which is much larger than all other *Mantophryne,* and has exceptionally high sexual‐size dimorphism. While an interrogation of the putative cryptic species complex within the *M. lateralis* clade is beyond the scope of our study, we find that a combination of tectonic rearrangement and climatic history are likely responsible for most of the diversity within the genus that we see today.

## Graphical abstract

Our dated phylogeny and biogeographic analysis of *Mantophryne*, a microhylid frog genus endemic to New Guinea, revealed origins in the East Papuan Composite Terrane (EPCT) in the late Pliocene. Dispersal and diversification out from the EPCT was driven by tectonic activity and climate reorganisation, creating habitat corridors and isolations in the Late Pliocene—Early Pleistocene. We recovered 13 *Mantophryne* clades that began to speciate ca. 8.5 million years ago, including eight distinct lineages currently assigned to *M. lateralis* , which started to diverge ca. 5.5 million years ago.

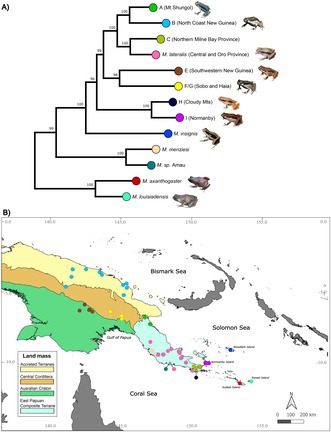

## 1 Introduction

Melanesia holds one of the richest anuran faunas in the world and supports the richest insular frog fauna, with almost 500 species known from New Guinea and associated land‐bridge islands (Oliver et al. 2022) and hundreds more known but awaiting description (Oliver et al. 2022; Ferreira et al. 2025). Its diversity has been facilitated by habitat isolation resulting from the orogeny of the Central Highlands as well as the generation of nearby islands either through crustal rifting or formation of volcanic islands. This is a direct consequence of the fact that New Guinea sits at the epicentre of the most tectonically active and complex region of the world—the junction where the Australian, Pacific, and Eurasian plates meet and created a series of interacting microplates (Hall 2002; Polhemus 2007). Within that junction are additional microplates that have become detached and moved independently of the three larger plates for extended periods (Bird 2003; Baldwin et al. 2012).

This tectonic activity has created a series of geological realms within Melanesia that have distinctively different origins and histories (e.g., Polhemus and Polhemus 1998; Polhemus and Allen 2007; Kraus 2021), leading to concomitantly isolated biotas that subsequently speciated. The East Papuan Composite Terrane (EPCT), which comprises the southeastern peninsula (Papuan Peninsula) of current New Guinea as well as its offshore islands, is of particular biological interest because of its status as a large and ancient stand‐alone island from at least the Oligocene until its recent suturing to the expanding Central Highlands (Pigram and Davies 1987; Quarles van Ufford and Cloos 2005). Hence, it has likely served as a long‐term source area for generating Melanesian biodiversity (e.g., Oliver et al. 2024), including a number of endemic genera.

Among the frog genera that originated in the EPCT is *Mantophryne* Boulenger 1897, a genus of medium‐sized terrestrial microhylid frogs that subsequently emigrated from the area in two separate dispersal events (Oliver et al. 2013). Oliver et al. (2013) generated their phylogenetic tree using three mitochondrial and three nuclear loci sampled for all four *Mantophryne* species known at that time, as well as several members of related microhylids as outgroups. Their recovered tree showed that one species of putative *Mantophryne* grouped instead with the closely related genus *Hylophorbus*, and that the monotypic genus *Pherohapsis* grouped within *Mantophryne*. They proposed the new nomenclature of *Hylophorbus infulatus* and *Mantophryne menziesi* . They also proposed that the most widely distributed species in this genus— *M. lateralis* —comprises nine molecularly divergent lineages that could be undescribed candidate species, though they took no formal taxonomic action on that result.

The study of Oliver et al. (2013) suggested the geographic origin and subsequent dispersal of *Mantophryne* was from the Papuan Peninsula. However, that study did not attempt to determine the timing of these events. Additional analyses are also required to determine the topological placement of the sole new species described since their work. Furthermore, their inferences of some of the biogeographic history of the genus were inconsistent with the then‐known geological history of that region. To address these issues, we undertook re‐analyses of Oliver et al. (2013)'s data, with the inclusion of more recently published sequence data, and newly generated data in the study, totalling 155 *Mantophryne* samples. We use these data to examine the biogeographic history of this genus in more detail. We also use available habitus data to explore patterns or outliers in body size between clades, and to quantify sexual size dimorphism of some members of the genus. Body size evolution of other New Guinea Microhylids has shown both phylogenetic conservatism and independent divergences correlated with ecological constraints (Rittmeyer et al. 2012; Oliver et al. 2017). Our exploration of habitus within *Mantophryne* alongside biogeography will therefore provide a baseline for further morphological investigation.

## 2 Methods

### 2.1 Sampling

In this study, we use data from 155 individuals representing the five currently described species of *Mantophryne*, of which 120 had previously published data and 35 samples were newly added by us to provide a more complete picture of their distribution. This includes 132 samples spanning the known distribution of the *M. lateralis* complex, five *M. louisiadensis* , five *M. axanthogaster* , three *M. insignis* , two *M. menziesi* , and eight specimens from a putatively undescribed species of *Mantophryne* from Amau (Oliver et al. 2013). All necessary research visas and export permits were granted by the National Research Institute and Department of Environment and Conservation of Papua New Guinea. We used two *Hylophorbus infulatus* samples—the sister genus to *Mantophryne*—as the outgroup for mitochondrial tree lineage determination. We provide detailed sample and locality information in Appendix S1. We also included further outgroup taxa for molecular‐dating analyses, accounting for available sequence data and calibration points, whilst limiting branch lengths between calibration points: *Chaperina fusca, Cophixalus balbus, Genyophryne thomsoni, Hylophorbus picoides, Kaloula picta, Oreophryne monticola*, and *Oreophryne brachypus* (Appendix S2). In total, we obtained 675 *Mantophryne* and 29 outgroup sequences from GenBank.

### 2.2 DNA Sequence Generation and Lineage Identification

We obtained ethanol‐preserved tissue samples from collections at Bishop Museum (BPBM), South Australian Museum (ABTC), and Michigan Museum of Zoology (UMMZ). We extracted whole genomic DNA from liver or muscle tissue using the Qiagen DNeasy Blood and Tissue kit (Qiagen, Hilden, Germany) in accordance with the manufacturer's instructions. We amplified and sequenced three mitochondrial gene regions (*12 s rRNA*, *16 s rRNA*, and *cytochrome b* (*cytb*)), and three nuclear loci (*c‐myc‐exon2 (cmycex2), c‐myc‐exon3 (cmycex3)*, and *tyrosinase1* (*tyr1*)). We amplified target DNA with PCRs in 25 μL reactions, consisting of 12.5 μL MyTaq Red Mix (Meridian Bioscience), 9.5 μL ultrapure water, 1 μL forward primer, 1 μL reverse primer (10 μM working concentration), and 1 μL DNA (standardised to ~25 ng/μL). We provide primer details and thermal cycling conditions in Appendices S3 and S4. We determined amplification success with gel electrophoresis using a 1% TAE agarose gel dyed with SYBR Safe gel stain and confirmed fragment size by comparisons with HyperLadder 50 bp. We prepared some amplicons using the Applied Biosystems Sanger Sequencing Kit and generated sequences using an Applied Biosystems 3500 Genetic Analyser. For other amplicons, we cleaned PCR products using ExoSAP‐IT (*Applied Biosystems*), which were then sequenced by DBS Genomics (Durham University) on an Applied Biosystems 3730 capillary instrument. We sequenced mitochondrial gene regions in the forward direction only, and nuclear loci in both directions.

We visualised raw sequence data in Geneious Prime v.2024.0.4 and manually refined chromatograms by trimming low‐quality base calls at the 5′ and 3′ ends of sequences, correcting miscalled bases, and assigning IUPAC codes to heterozygous positions in nuDNA sequences. No unexpected stop codons were detected in protein‐coding sequences. Sequences from our project are deposited in NCBI (newly generated sequence accessions detailed in Appendix S1) [to be included upon manuscript acceptance]. We aligned our newly generated sequences with existing data available through NCBI, in Geneious Prime using the Clustal Omega algorithm v.1.2 (Sievers et al. 2011). We generated a maximum‐likelihood phylogeny using concatenated mtDNA loci on the IQ‐TREE Web Server v.1.6.12 (Trifinopoulos et al. 2016) with ModelFinder + tree reconstruction +1000 ultrafast bootstrap replicates, rooted to *H. infulatus* . The best‐fit model chosen according to Bayesian Information Criterion (BIC) in IQ‐TREE was TPM2u + F + I + G4. We visually inspected the phylogeny in FigTree v.1.4.4 (Rambaut 2009), and assessed pairwise between‐group distances of lineages for mtDNA alignments in MEGA11 v.11.0.13 (Tamura et al. 2021). We considered lineages to be “true” for further analyses when *p*‐distance was > 4% sequence divergent in *cytb* (Appendix S5).

**FIGURE 1:**
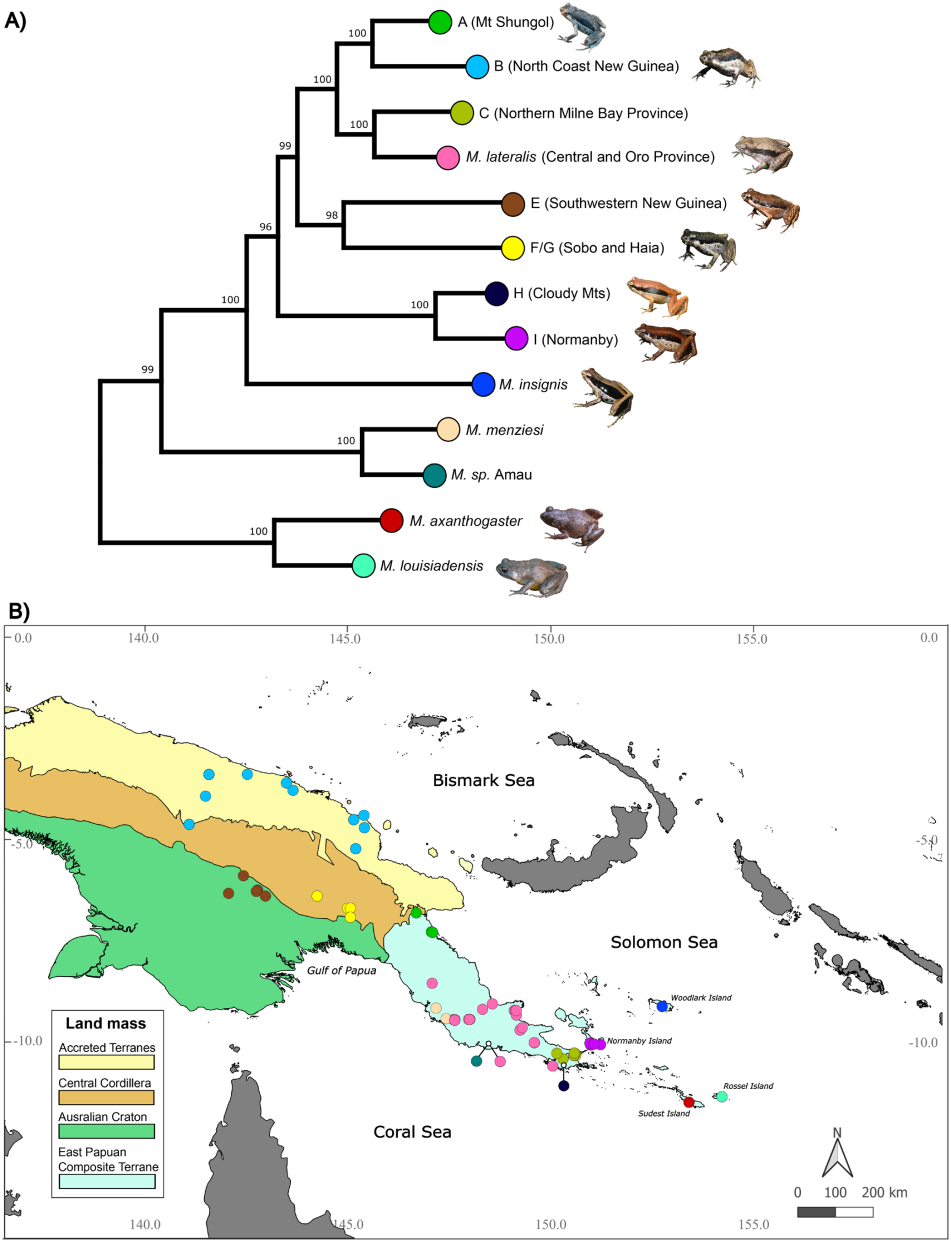
(A) Concatenated mitochondrial maximum‐likelihood (ML) phylogeny. ML support values for each node are given above branches. Where available, images of representative species for named lineages are presented alongside branch tips. (B) Distribution map of all *Mantophryne* lineages on the four major land masses that comprise New Guinea. Colours indicate species and *M. lateralis* clades detailed in the ML tree in (A). Dots representing *M*. sp. Amau + *M. lateralis sensu stricto* are at the same locality in Amau, and Clade H (Cloudy Mts) is set off from the map with lines indicating specific localities for clarity where there is considerable overlap in points on the map. *M. lateralis* refers to *M. lateralis sensu stricto* in the main text.

We plotted lineages using available coordinates in QGIS v.3.34, with layers for the four major land masses (Figure 1B): (1) the Central Highlands (CH), which is the mountainous spine of New Guinea west of the Papuan Peninsula; (2) Accreted Terranes (AccTerr), which incorporates the mountainous part of New Guinea north of the Central Highlands, extending from the Huon Peninsula west through the Adelbert, Torricelli, Prince Alexander and Bewani mountains; (3) the Australian Craton (AusCrat), comprising the lowlands south of the Central Highlands; and (4) the East Papuan Composite Terrane (EPCT), which includes the Papuan Peninsula east of the Purari Basin and south of Lae and all its offshore islands.

We used PHASE v.2.1.1 (Stephens et al. 2001) to determine haplotypes in heterozygous positions in nuclear loci. PHASE manages heterozygous positions by statistically inferring haplotypes using linkage disequilibrium, recombination, and population‐level data to make probabilistic estimates of the correct haplotypes, with the output including both the phased haplotypes. We first prepared files for phasing using seqPHASE (Flot 2010). We used a recombination model (−d1), a random seed, an 80% threshold for posterior probabilities (−p0.8), and ran the analyses for 1000 iterations with a thinning interval of 10 and burn‐in of 100. We then constructed median‐joining haplotype networks for each nuDNA locus in PopART v.1.7 (Leigh et al. 2015) to further visualise ancestral gene networks and distribution of mitochondrial clades in the nuclear data.

### 2.3 Divergence‐Date Estimation and Biogeography

We estimated phylogenetic relationships with a Bayesian multispecies coalescent approach by generating species‐trees in *BEAST (Heled and Drummond 2009); files were prepared using BEAUTi2 (Drummond et al. 2012). We used alignments for *cytb, cmycex2, cmycex3*, and *tyr1*; we did not use alignments for 12 s and 16 s due to convergence issues, as well as to reduce our preliminary extensive outgroup selection. We initially configured site models for each locus using best‐fit models generated by IQ‐TREE v.1.6.12; however, the species tree did not converge on these models nor on simplified models. We therefore determined site models using the internal BEAST model test. After initial tests applying relaxed lognormal clocks, we determined that strict clocks for each partition were the most appropriate to use with the data. We used a calibrated Yule model with a random starting tree for each locus and applied three secondary calibration priors with normal distributions, obtained from divergence‐date estimates from a combination of fossil‐calibrated trees generated by Brennan et al. (2024) and Portik et al. (2023). Most‐recent common ancestor (MRCA) calibrations were as follows: (1) *Mantophryne* and *Hylophorbus*, with a mean of 14.7 million years ago (MYA), and sigma of 2.2 (19–10.4 MYA); (2) *Genyophryne, Oreophryne* and *Cophixalus*, with a mean of 21.26 MYA, and sigma 1.87 (24.9–17.6 MYA); and (3) *Chaperina* and *Kaloula*, with a mean of 36.5 MYA, and sigma of 4.0 (44.3–28.7 MYA). We ran the analyses for 300,000,000 generations, with sampling every 50,000 generations; the first 20% of trees were discarded as burn‐in. We analysed the XML files from BEAUTi2 with BEAST2 (Bouckaert et al. 2014) on the CIPRES Science Gateway v.3.3 (Miller et al. 2010) platform. We assessed convergence using Tracer v.1.7 (Rambaut et al. 2018), with successful convergence determined if good mixing of the priors was observed and ESS ≥ 200 was achieved.

To assess the stability of taxon placement across the posterior distribution of trees we used the *R* package *Rogue* v.1.0 (Smith 2022) on the output from *BEAST, discarding the first 10% of trees as burn‐in and using the QuickRogue() and RogueTaxa() functions to identify possible rogue taxa. We calculated tip instability and tip volatility using the TipInstability() and TipVolatility() functions, respectively. Upon identification of a clade that was causing tip instability and volatility, we re‐ran a *BEAST analysis with that taxon removed.

To estimate biogeographic history, we assigned lineages to one of the four geological regions detailed above. We estimated ancestral geographic areas for the original dated species tree after outgroups were pruned from the tree using the drop. tip command in the R package *ape* (Paradis and Schliep 2019). We implemented the dispersal‐extinction‐cladogenesis (DEC; Ree et al. 2005, Ree and Smith 2008) and dispersal‐vicariance analysis (DIVALIKE; Ronquist 1997) models, alongside their respective ‘jump dispersal’ parameters in BioGeoBEARS v.1.1 (Matzke 2014) in RASP v.4.4 (Yu et al. 2015). We determined the model with the best fit using Akaike information criterion (AIC) scores and weights.

### 2.4 Habitus

We have SVL and mass information for 56 of the *Mantophryne* specimens used in our biogeographic analyses, and we used these data to assess body size evolution between clades. We assigned an additional 147 *Mantophryne* specimens previously collected by F. Kraus with available SVL and mass information to respective clades using locality information, for a total of 203 individuals of all sexes and sizes (Appendix S9). We euthanised animals with MS222 and measured snout‐vent length (SVL) to the nearest 0.1 mm with callipers; we measured mass at the same time with either a 10‐g (to the nearest 0.1 g) or 60‐g (to the nearest 0.5 g) Pesola scale, depending on the size of the animal. Animals were then fixed in formalin and deposited in Bernice P. Bishop Museum, Honolulu, Hawaii, for long‐term storage in 70% ethanol. We did not have both SVL and mass information for *M. menziesi* or *M. lateralis* clades E, F, and G, and Clade A was only represented by one specimen. For each clade, we plotted SVL and mass in the *R* package *ggplot2* v.3.5.2 (Wickham 2016) and ran a power regression for the relationship in Microsoft Excel.

Sex information was also available for *M. axanthogaster* (18 M, 12 F) and *M. louisiadensis* (4 M, 22 F), so for those species, we plotted SVL and mass separately by sex to assess presence of sexual‐size dimorphism. To understand sexual‐size dimorphism, we used the sexual‐dimorphism index (SDI) (Lovich and Gibbons 1992), SDI = (L/S)−1, for which L is the average size of the larger sex of the species (in this study entirely females), and S is the average size of the smaller sex.

## 3 Results

We generated an ML tree of 155 *Mantophryne* and two *Hylophorbus infulatus* samples consisting of 1592 bp of mitochondrial sequence data (669 bp for *12 s*, 435 bp for *16 s*, and 435 bp for *cytb*), identifying 13 discrete lineages with well‐supported nodal support (Figure 1A; Appendix S6). A clade consisting of *M. louisiadensis* and *M. axanthogaster* is sister to the remaining *Mantophryne*, and a clade consisting of *M. menziesi* and *M*. sp. Amau is sister to *M. insignis* and *M. lateralis sensu lato*. We identified eight distinct sublineages within *M. lateralis sensu lato* (A—I) with > 4% sequence divergence in *cytb*; we follow the coding system for *M. lateralis* lineages used by Oliver et al. (2013). *Mantophryne lateralis* clades B and I had the highest divergence, at 13.83%, whereas *M. lateralis* clades H and I had the lowest divergence, at 4.72% in *cytb* (Appendix S5). We consider *M. lateralis* Clade D to be *Mantophryne lateralis* *sensu stricto* because it is the only clade of that species in the vicinity of the type locality. All but three *M. lateralis* clades (B, E, F) occur within the EPCT (Figure 1B).

**FIGURE 2:**
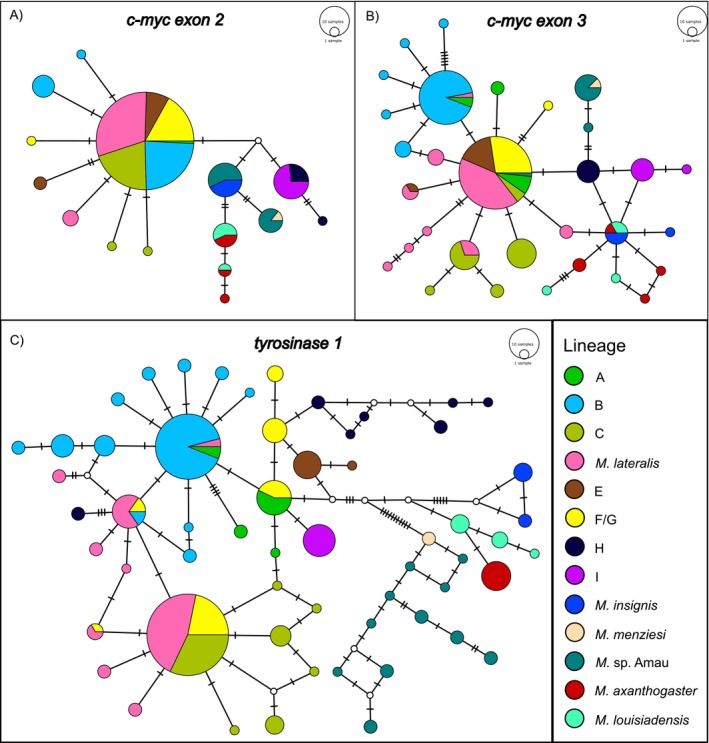
Median‐joining haplotype networks constructed in PopART for nuclear loci: (A) *c‐myc* exon 2, (B) c*‐myc* exon 3, and (C) *tyrosinase 1*. Haplotypes are represented by circles whose colours denote lineages, and sizes are proportional to the number of alleles. Hatches on connecting lines indicate the number of mutational steps, and inferred but missing haplotypes are represented by small white circles.

Nuclear haplotype networks show varying levels of clustering of identified mitochondrial clades (Figure 2). *cmycex2* and *cmycex3* have less allelic variation and consequently more shared haplotypes among clades. A widespread, likely ancestral, *M. lateralis sensu lato* haplotype exists for *M. lateralis sensu stricto* + clades A—F/G (clade F of Oliver et al. (2013) = their clade G, see below) in both *cmycex2* and *cmycex3*. In *cmycex2* and *cmycex3*, *M. menziesi* has no unique haplotypes and shares the single haplotype it has at each locus with *M*. sp. Amau. Additionally, all samples of *M. insignis* share a haplotype with the second haplotype containing *M*. sp. Amau in *cmycex2*. All *M. lateralis* Clade I samples share a single haplotype with samples belonging to *M. lateralis* Clade H, and the two *M. louisiadensis* haplotypes are shared with *M. axanthogaster* samples in *cmycex2*. We recovered at least one unique haplotype at cmycex2 and *cmycex3* for all other identified clades.

At least one unique haplotype in our *tyr1* network was identified for all mitochondrial clades (Figure 2C). *Mantophryne insignis, M. louisiadensis, M. axanthogaster, M. menziesi, M*. sp. Amau, and *M. lateralis* clades E, H, and I share no haplotypes with any other clade. *Mantophryne menziesi* and *M*. sp. Amau differ by a minimum of one mutational step from one another, as do *M. louisiadensis* and *M. axanthogaster* . Our samples of *M. lateralis* Clade H appear in two distinct parts of the network, differing by a minimum of seven mutational steps.

**FIGURE 3:**
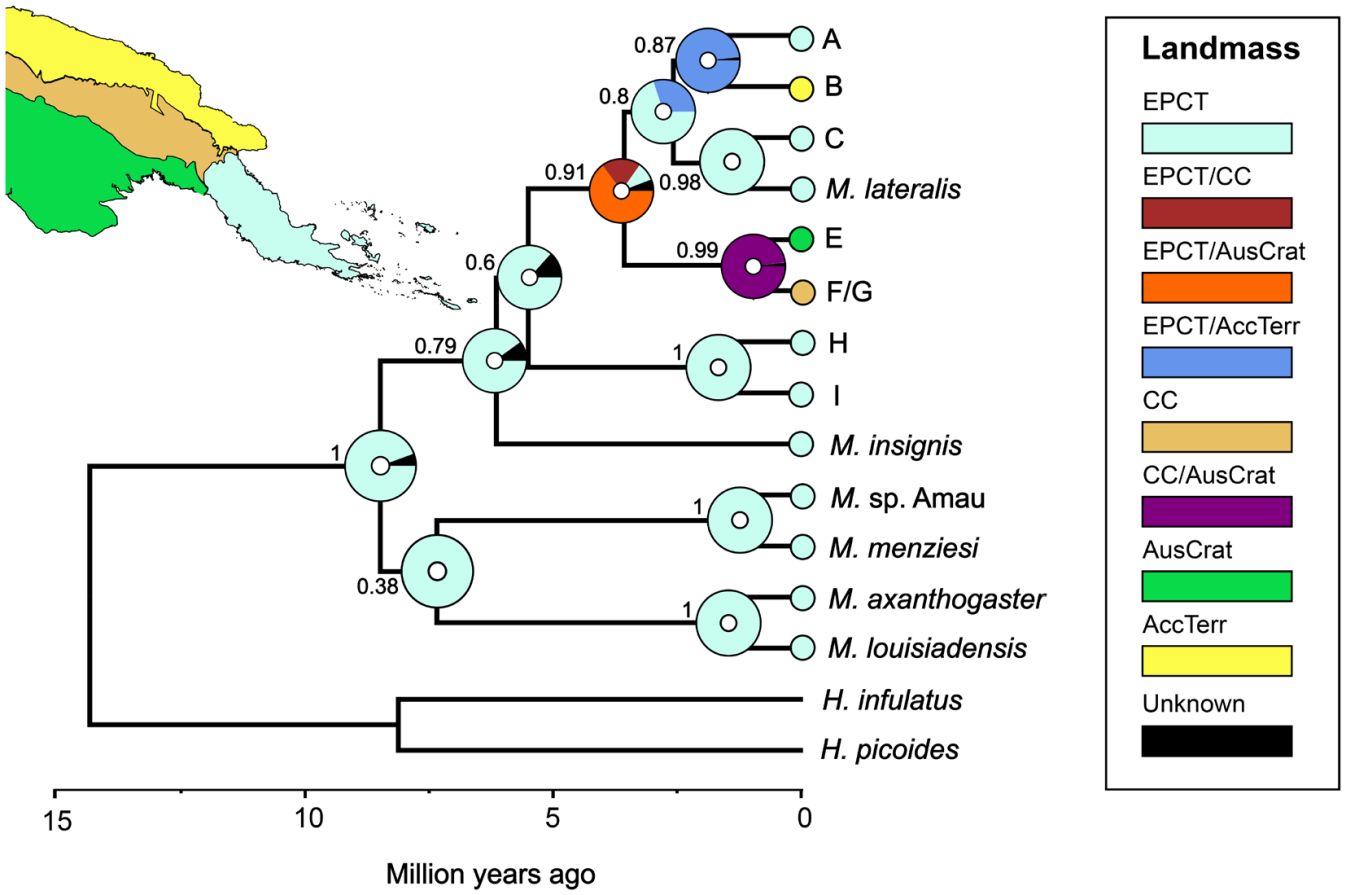
Time‐calibrated phylogeny generated using *BEAST with ancestral geographic ranges estimated by BioGeoBEARS. Single geological regions are illustrated by coloured regions in a partial map of New Guinea, and inferred ancestral ranges involving two geological regions are denoted by unique colours (see key). Support values at each node are Bayesian posterior probabilities.

Our dated species tree (Figure 3; Appendix S7) consisted of 1695 bp of sequence data (489 bp for *cytb*, 347 bp for *cmycex2*, 356 bp for *cmycex3*, and 503 bp for *tyr1*). The best‐fitting model for the biogeographic reconstructions was the Dispersal‐Extinction‐Cladogenesis (DEC) model with an AICc score of 29.78, closely followed by BAYAREALIKE+J (AICc 30.1) (Appendix S8). Many of the internal nodes of the species tree are poorly supported (Figure 3). The two fully supported clades comprising *M. louisiadensis* + *M. axanthogaster* and *M. menziesi* + *M*. sp. Amau appear sister to each other but lack statistical support (0.38 Bayesian posterior probability [bpp]). *Mantophryne insignis* and *M. lateralis sensu lato* have cladal statistical support of 0.79 bpp. Within the *M. lateralis sensu lato* clade, there is no support for the sister relationship between *M. lateralis* clades H + I to the remaining *M. lateralis* clades (0.6 bpp). We found that *Mantophryne* and *Hylophorbus* likely diverged ca. 14.4 MYA (17.6–11.4 MYA 95% HPD), with *Mantophryne* starting to speciate ca. 8.5 MYA (11.2–6 MYA 95% HPD) with high probability (95% likelihood) in the East Papuan Composite Terrane (EPCT) (Appendix S7). *Mantophryne lateralis sensu lato* started to diversify ca. 5.5 MYA (7.4–4 MYA 95% HPD), also within the EPCT. The ancestor of each clade was inferred to be from the EPCT or to be shared between the EPCT and another region, except for the ancestor of *M. lateralis* clades E and F, which is estimated to have diverged ca. 0.9 MYA (2.2–0.26 MYA 95% HPD) within the area of the Australian Craton and Central Highlands (99% likelihood). The ancestor of *M. lateralis* clades A—F/G + *M. lateralis sensu stricto* diverged ca. 3.6 MYA (5.2–2.2 MYA 95% HPD) and had a 65% chance of being from the EPCT and Australian Craton. The ancestor of *M. lateralis* clades A and B had a 99% chance of being from the EPCT and Accreted Terranes. All pairs of *M. lateralis* sister clades diverged from one another 2.7–0.3 MYA (95% HPD limits).

Due to poor support in the species tree, we tested for rogue taxa and tip instability and volatility. The rogue‐taxon‐specific analysis did not identify any clades as being rogue. However, in both tip instability and volatility, *M. insignis* received the highest values for both instability (0.07159) and volatility (0.02547), suggesting the possibility that the species is a rogue taxon in our dataset. Following re‐analysis of the species tree upon removal of *M. insignis* , relationships within *Mantophryne* became well resolved, with *M. louisiadensis* + *M. axanthogaster* being sister to the remaining clades, and then *M. menziesi* + *M*. sp. Amau resolved as sister to *M. lateralis sensu lato*.

**FIGURE 4:**
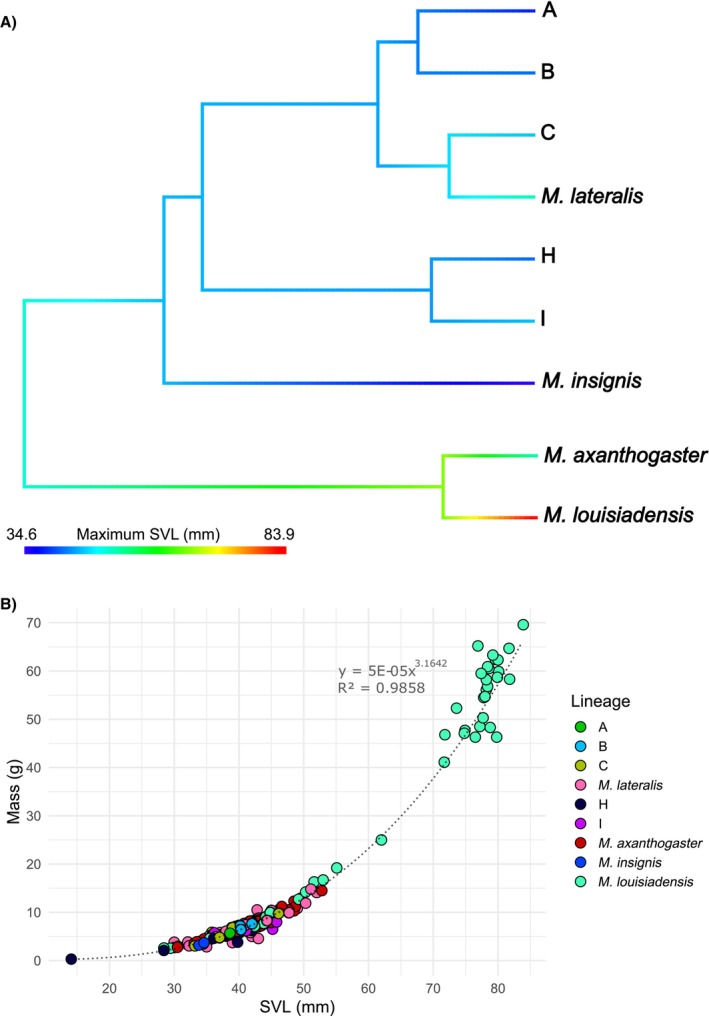
(A) Evolution of body size in *Mantophryne* with available snout‐vent‐length (SVL) data, using our time‐calibrated phylogeny. Branch colours represent maximum SVL and colour gradient indicates the SVL range of *Mantophryne* in our dataset. (B) Changes in mass with increasing snout‐vent length (SVL) shown for most lineages. Coloured dots represent lineages for which data are available; trendline is a power regression. Regression equation (y) and *R* ^2^ values are given.

**FIGURE 5:**
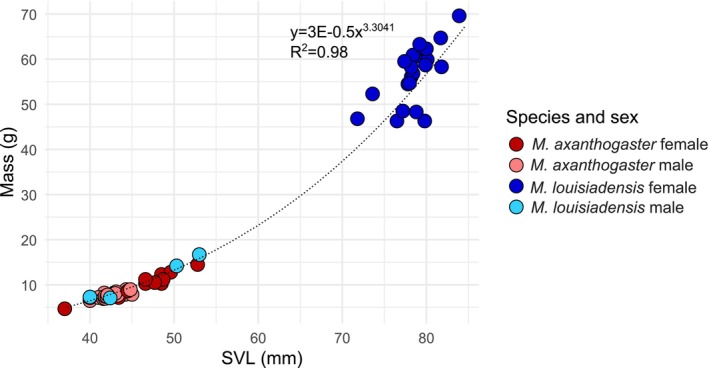
Mass vs. snout‐vent length (SVL) for *M. axanthogaster* and *M. louisiadensis* , showing sexual size dimorphism in the latter. Coloured dots indicate species and sex with a power trendline. Trendline equation (y) and *R* ^2^ values are given.

*Mantophryne* demonstrates morphological conservatism for SVL (Figure 4a), including a range of just 13.4 mm in maximum SVL within all *M. lateralis* clades (Appendix S9). Variation in maximum and mean SVL within sister pairs is minimal, with the exception of *M. louisiadensis and M. axanthogaster*, driven by relatively large females in *M. louisiadensis* (Figure 5; Appendix S9). All lineages of *Mantophryne* for which SVL and mass data are available increase their mass in a power relationship of 3.16 to SVL, with most variation in mass being explained by SVL (Figure 4b). Among these species, *M. axanthogaster* and especially *M. louisiadensis* are the largest in body size (Figure 5). Sexualsize dimorphism in *M. axanthogaster* (SDI = 0.099 for SVL, 0.312 for mass) is, however, unexceptional among asterophryine frogs whereas that for *M. louisiadensis* is among the more extreme known (Figure 5; SDI = 0.686 for SVL, 4.023 for mass).

## 4 Discussion

Our findings add to the growing body of evidence that the East Papuan Composite Terrane (EPCT) has given rise to high levels of endemicity, making it one of the most densely biodiverse regions of the world for herpetofauna (Kraus 2021). Our results are largely congruent with those of Oliver et al. (2013) who concluded that other regions of New Guinea occupied by *Mantophryne* were colonised from source populations in the EPCT. However, our study adds dates to these origin and dispersal events, and we provide improved geographic coverage and clade support. *Mantophryne* is a fairly recent lineage, having diverged from *Hylophorbus* in the mid‐Miocene and radiated since the late Miocene, with most of that divergence occurring in the Pleistocene. *Mantophryne axanthogaster* and *M. louisiadensis* are sister taxa, as are *M. menziesi* and *M*. sp. Amau, and these two species pairs functionally form a polytomy with the remaining *Mantophryne* lineages when analysed in a dataset that includes *M. insignis* (Figure 3). Early divergence among these three clades separates *M. menziesi* + *M*. sp. Amau in Central Province from those ancestral lineages occurring on the north side of the Owen Stanleys or south of the Musa Divide, a low‐elevation area in the Owen Stanley Range that separates the Milne Bay Region (*sensu* Kraus 2021) from regions to the northwest. We propose that these three areas became isolated from one another during the late Miocene as climate cooled and dried (Herbert et al. 2016; Scotese et al. 2021) sufficiently to transform the Musa Divide into savannah. This ecological change would have isolated the three regions simultaneously, producing the polytomy seen in our tree.

The putative *M*. sp. Amau species split < 3 MYA (2.6–0.3 MYA 95% HPD) from *M. menziesi* on the southern coastal region of the EPCT,Iex and this may be indicative of vicariance since both lineages occur at < 150 m elevation and are separated in our sampling by a distance of approximately 150 km of interspersed hilly terrain. Within the Milne Bay Region, the two species on the Louisiade Islands diverged from the remaining lineages presumably as rifting of the Woodlark Basin caused the intervening Pocklington Rise to submerge, isolating those islands from the mainland, with which they were previously contiguous (Taylor et al. 1995, 1999; Miller et al. 2012). The divergence of *M. axanthogaster* and *M. louisiadensis* from each other is relatively recent (3–0.5 MYA 95% HPD), likely reflecting their genetic continuity until continued crustal relaxation generated by further opening of the Woodlark Rift finally separated those islands from each other. The divergence of *M. insignis* 8.3–4.6 MYA (95% HPD) from the remaining lineages also stemmed from continued sundering of the Milne Bay Region by the opening of the Woodlark Rift, occurring after the submergence of the Pocklington Rise but before the separation of Sudest from Rossel Island. The next‐most‐proximate divergences in the genus still involve lineages confined to Milne Bay Province ( *M. lateralis* clades H and I). It wasn't until ca. 7.4–4 MYA (95% HPD) that some of the clades of *M. lateralis sensu lato* dispersed from the Milne Bay Region and crossed into the more westerly regions of the Papuan Peninsula, where one lineage ( *M. lateralis* Clade C) later crossed back into Milne Bay Province. Another dispersal involving the ancestor of *M. lateralis* Clade E and Clade F/G occurred from the Papuan Peninsula into the Central Highlands and Australian Craton 2.6–0.9 MYA (95% HPD), and one more lineage ( *M. lateralis* Clade B) spread across the northern versant of New Guinea west of the Papuan Peninsula.

*Mantophryne lateralis sensu stricto* + *M. lateralis* Clade C split from *M. lateralis* Clade A + B in the mid‐late Pliocene 3.5–1.8 MYA (95% HPD) when *M. lateralis sensu stricto* then occupied a large area north and south of the Owen Stanley mountains and *M. lateralis* Clade C clustered in eastern Milne Bay Province. The dispersal age and pattern of *M. lateralis sensu stricto* may suggest a southeastern EPCT origin or dispersal around the low‐elevation Musa Divide. Although *M. lateralis* clades A and B form a sister pair, Clade A remains within the far northeastern limit of the EPCT, and Clade B extends into the Accreted Terranes from the Finisterre Mts. and Markham River westward; it now represents the most widely distributed of the *Mantophryne* lineages. This split occurred 2.7–1.1 MYA (95% HPD), relatively soon after the smoothly continuous collision of the Finisterre Block with New Guinea from 6 to 3 MYA (Abbott 1995) resulted in a subaerial land connection with New Guinea ca. 1.3 MYA (Abbott et al. 1994). Continued uplift of the Finisterre Terrane could have provided the isolation needed to genetically separate these two clades.

Sister *M. lateralis* clades H and I diverged from all other *M. lateralis* lineages 8–3 MYA and then diverged from each other 3–1 MYA, when Clade H likely evolved parapatrically with clades D and C, and Clade I became geographically isolated on Normanby Island. Biogeographic connection between Normanby Island and southeastern‐most mainland New Guinea has been noted for a variety of herpetofaunal species (Kraus 2021), presumably reflecting a closer geographic relationship between the two areas in the past. Like the other D'Entrecasteaux islands, Normanby is composed in part of metamorphic core complex exhumed with crustal extension caused by the opening of the Woodlark Rift (Baldwin et al. 1993, 2012; Hill and Baldwin 1993). Consequently, the island is young, and its derivation from crustal extension during the expansion of the Woodlark Basin explains why the split between *M. lateralis* clades H and I is more or less of the same age as that between *M. axanthogaster* and *M. louisiadensis* , which also became isolated due to the active expansion of the Woodlark Basin.

A striking feature of the six recent pairs of sister taxa within *Mantophryne* (clades A + B, C + *M. lateralis sensu stricto*, Clade E + Clade F/G, Clade H + Clade I, *M*. sp. Amau + *M. menziesi* , *M. axanthogaster* + *M. louisiadensis* ) is that five of them appear to have diverged more or less simultaneously during the late Pliocene—early Pleistocene (3–1 MYA) (Figure 3; Appendix S7). This period coincides with a significant climatic reorganisation, likely triggered by tectonic activity modifying the Indonesian Passages (Krebs et al. 2011; Christensen et al. 2017). These changes included the constriction of passages between the Bird's Head Peninsula (western New Guinea) and Sulawesi, the uplift of Halmahera, and the collision of the Australian Plate with the Banda Arc, causing deformation and uplift (Cane and Molnar 2001; Krebs et al. 2011). This geological reconfiguration restricted the Indonesian Throughflow (ITF) current, leading to a switch of flow from warm South Equatorial Pacific waters to relatively cool North Equatorial Pacific waters (Cane and Molnar 2001).

This change, along with reduced volume transport and increased upwelling, likely caused sea‐surface temperature cooling (up to 2°C–3°C cooler) in the eastern Indian Ocean. This geological rearrangement increased sea‐surface temperatures in the Pacific Warm Pool (0.9°C warmer) because less heat was exported to the Indian Ocean, and this led to an anomalous precipitation dipole across the Indonesian Passages, likely resulting in increased rainfall across much of New Guinea and Indonesia and decreased precipitation in the eastern Indian Ocean (Krebs et al. 2011). This led to the aridification of Australia, with precipitation dropping substantially over most areas (Martin 2006; Fujioka and Chappell 2010; Krebs et al. 2011; Christensen et al. 2017).

Western Melanesia would have been at a critical juncture between aridification and high rainfall at this time. It is possible that southern New Guinea, being closer to Australia, experienced drying of rainforests and expansion of savannahs, evidenced by rainforest contractions in northeast Australia (see Bryant and Krosch 2016) and the presence of isolated savannahs in the Milne Bay region to the present day. Areas to the north of the Central Cordillera likely experienced increased precipitation, being in proximity to the Pacific Warm Pool. This plausible north–south precipitation gradient could have created asynchronous habitat changes, leading to isolation of populations, and this coincides with the consistent divergence dates of our *Mantophryne* sister‐taxon splits.

An additional biogeographic issue requires comment. Oliver et al. (2013: 606) claimed that colonisation of the Louisiade and D'Entrecasteaux islands by *Mantophryne* required two overwater dispersal events from New Guinea. However, that hypothesis is implausible, because the Louisiade Islands were connected to New Guinea by continuous land until the opening of the Woodlark Rift and spreading of the Woodlark Basin caused the intervening land to subside beneath the sea, isolating the terminus of the Owen Stanley Range as the Louisiade Islands (Pigram and Davies 1987; Taylor et al. 1995, 1999; Miller et al. 2010). As we stated above, this submergence of the Pocklington Rise is certainly what led to the initial isolation of the clade [ *M. axanthogaster* + *M. louisiadensis* ], and so no trans‐marine dispersal was necessary. Similarly, although the mechanism is less certain, the presence of multiple frog genera (32 species in 14 genera) on the D'Entrecasteaux Islands almost certainly involved overland dispersal because independent trans‐marine dispersal of so many salt‐intolerant lineages would be highly unlikely (Kraus 2015). This may have involved either a dry‐land connection of the D'Entrecasteaux Islands to New Guinea before the continued opening of the Woodlark Rift sundering that connection, or it may have involved a land connection during one of the glacial periods, when sea levels were much lower. In any event, independent trans‐marine dispersal of so many frogs to the nearby D'Entrecasteaux Islands seems highly unlikely, casting doubt on *Mantophryne* having arrived there in that manner.

Our interrogation of the sequence data of *M. lateralis* clades F and G in Oliver et al. (2013) revealed that there was almost certainly a labelling error in either the laboratory methods or at the data‐handling stage for sample ABTC 42829 from Haia, Chimbu Province (the sole individual of their Clade F). In *cytb* and *16 s*, ABTC 42829 is identical to a sample from the same locality (ABTC 42858) that they assigned to Clade G. However, in the *12 s* dataset, ABTC 42829 clusters with samples in Clade B. Because mitochondrial genes are all linked, the placement of the same sample in two different locations in comparable phylogenetic trees based only on mtDNA genes would not be possible, and thus, we determine that the *12 s* sequence is erroneous. When we removed *12 s* from analyses, we found no support for Oliver et al.'s Clade F. Thus, we deleted it as a separate lineage, and we have referred to these frogs as “Clade F/G” above. Further, whilst we are unable to conclusively test for this with the data to hand, it is also possible that the *tyr1* sequence for BPBM 15411 from the Cloudy Mountains, Milne Bay Province, has been mislabelled since it occupies haplotype network space a long way from other members of its assigned clade (Clade H).

We find evidence of mito‐nuclear discordance and discordance among nuclear genes within *Mantophryne*. *Mantophryne insignis* is sister to *M. lateralis* *sensu lato* in the mitochondrial tree, but in the nuclear loci that species consistently clusters with other taxa: in *cmycex2* it shares a haplotype with *M*. sp. Amau, in *cmycex3* it shares a haplotype with *M. louisiadensis* and *M. axanthogaster* in most of the individuals but with one additional sample being a single mutational step away the remaining samples, and in the faster‐evolving *tyr1* the species does not share any haplotypes, but the fewest mutational steps are to a group containing *M. louisiadensis* and *M. axanthogaster* (Figures 1, 2, 3). In *cmycex3*, the haplotype cluster containing *M. menziesi* and *M*. sp. Amau is separated from *M. louisiadensis* and *M. axanthogaster* by a haplotype containing all *M. lateralis* Clade H samples. These locus‐specific differences explain why there is poor support within several of the non‐sister lineages in both Oliver et al. (2013) and in our species tree, with many of the early splits in the tree being polytomies. Exploration of our species tree revealed that *M. insignis* causes tip instability and volatility. Rerunning our species tree with *M. insignis* removed produced a well‐supported topology in which *M. menziesi* + *M*. sp. Amau are sister to *M. lateralis sensu lato*, as was reported in Oliver et al. (2013). It seems likely that the discordant clustering of *M. insignis* with congenerics in the gene networks is indicative of a true polytomy whereby vicariance events separated major clades during the same time, likely during the opening of the Woodlark Rift. The apparent resolution of these basal relationships seen in Oliver et al. (2013) and in our tree when *M. insignis* is removed is likely an artefact of incomplete lineage sampling, which, of course, could not have been addressed by Oliver et al. (2013), which was published before *M. insignis* was known to science.

Morphological evolution in size and SSD in *Mantophryne* has been considerable considering the few species and the relatively short time period involved. However, we had insufficient data to examine this issue across the genus in detail. Our results suggest that all species likely fall along the same ontogenetic trajectory for mass gain with increasing length (Figure 4b), and we demonstrated that the large outlying size of *M. louisiadensis* is a phenomenon of high sexual‐size dimorphism with females of that species being notably larger than males (Figure 5). We were unable to quantify the short, squat habitus of *M. menziesi* nor the gracile status of *M. insignis* due to unavailable or limited morphological data, though those features are clear from their original descriptions (Zweifel 1972; Günther and Richards 2016) or from direct examination of animals in life (Kraus, pers. obs.). All lineages currently assigned to *M. lateralis* , however, are of intermediate build to these (Zweifel 1972; Richards 2025; Kraus, pers. obs.) and show no obvious differences to each other in habitus (Figure 4a). Further work will be needed to relate these morphological differences to each species' ecology, though it is clear that the gracile habitus of *M. insignis* is what allows that species to climb up to 4 m above the ground (Günther and Richards 2016), the only *Mantophryne* species to engage in scansorial behaviour (Zweifel 1972; Richards 2025; Kraus, pers. obs.). The island rule, a phenomenon whereby species/populations will experience gigantism or dwarfism on islands, could offer an explanation for the habitus that we see within the genus. The island rule seems to be less common in amphibians compared to other vertebrates, where most evidence leans weakly towards gigantism (e.g., Benítez‐López et al. 2021), but this pattern somewhat matches with patterns observed in the *M. louisiadensis* and *M. axanthogaster* clade.

One final note is that although we recognise eight genetic lineages within what is currently called *Mantophryne lateralis* , like Oliver et al. (2013) we choose not to speculate as to whether these lineages represent distinct species. To do so requires additional data from morphology and especially the structure of advertisement calls, examinations that are beyond the scope of this study.

## Author Contributions

**Rebecca S. Morris:** data curation (lead), formal analysis (equal), investigation (equal), methodology (equal), project administration (equal), visualization (equal), writing – original draft (equal), writing – review and editing (equal). **Fred Kraus:** conceptualization (equal), formal analysis (equal), funding acquisition (equal), methodology (equal), project administration (equal), resources (equal), writing – original draft (equal), writing – review and editing (equal). **V. Deepak:** formal analysis (equal), methodology (equal), visualization (equal), writing – original draft (equal), writing – review and editing (equal). **Saunak Pal:** formal analysis (equal), methodology (equal), visualization (equal), writing – original draft (equal), writing – review and editing (equal). **Stephen J. Richards:** funding acquisition (equal), methodology (equal), resources (equal), writing – original draft (equal), writing – review and editing (equal). **Simon T. Maddock:** conceptualization (equal), formal analysis (equal), funding acquisition (equal), methodology (equal), project administration (equal), resources (equal), supervision (lead), visualization (equal), writing – original draft (equal), writing – review and editing (equal).

## Funding

This work was supported by the National Science Foundation (DEB‐0103794), DEB‐0743890, DEB‐2230919, Conservation International, and Natural Environment Research Council (NE/W006774/1).

## Disclosure

The authors have nothing to report.

## Conflicts of Interest

The authors declare no conflicts of interest.

## Supporting information


**Appendix S1:** Catalogue numbers, species, locality and GenBank sequence accession numbers by locus for each specimen used for molecular analyses.

**Appendix S2:** Species and GenBank accession numbers of outgroups used for BEAST analyses.

**Appendix S3:** Loci, primer names and primer sequences in forward and reverse directions.

**Appendix S4:** Optimised thermal‐cycling conditions for each locus.

**Appendix S5:** Pairwise p‐distance matrix for nucleotide differences between lineages in cytochrome‐B (cytb), produced in MEGA11 using the between group mean‐distance function.

**Appendix S6:** Full maximum‐likelihood (ML) phylogeny using concatenated mitochondrial loci generated on the IQTREE Web Server. Museum catalogue numbers and localities are given at each tip. Sequences from samples added to this study in addition to those used by Oliver et al. (2013) are indicated by italics. ML support values for each node are given above branches. Lineages were allocated based on *cytb p*‐values > 4% and followed the lettering of Oliver et al. (2013). After we interrogated the sequence data for specimen ABTC42829, previously identified by Oliver et al. (2013) as a unique lineage ( *M. lateralis* Clade F), we determined a labelling or sequence error must have occurred in 12 s and, using data only for 16 s and *cytb*, found this specimen to fall within Clade G; we therefore created Clade F/G to remain consistent with the lettering used by Oliver et al. (2013).

**Appendix S7:** Time‐calibrated phylogeny of *Mantophryne* and outgroups. Scale bar indicates millions of years ago (MYA) and error bars at node are maximum and minimum estimates for divergence dates.

**Appendix S8:** Best‐fit model selection test output from BioGeoBEARS.

**Appendix S9:** Catalogue number/collection ID, lineage, locality, habitus and sex information for each specimen used for habitus analyses.

## Data Availability

All new sequence data will be deposited in GenBank upon manuscript acceptance; all other data not included in the manuscript or appendices will be deposited in figshare at https://figshare.com/projects/Article_1/267656.
