# Supplementary figures and images for: Genetic Diversification in a New Guinean Frog Genus (Mantophryne, Microhylidae) was Driven by Ancient Tectonic Activity and Climate Reorganisation
Source: Ecol Evol. 2026 May 6;16(5):e73291. doi: 10.1002/ece3.73291 (PMC13148145; doi:10.1002/ece3.73291)

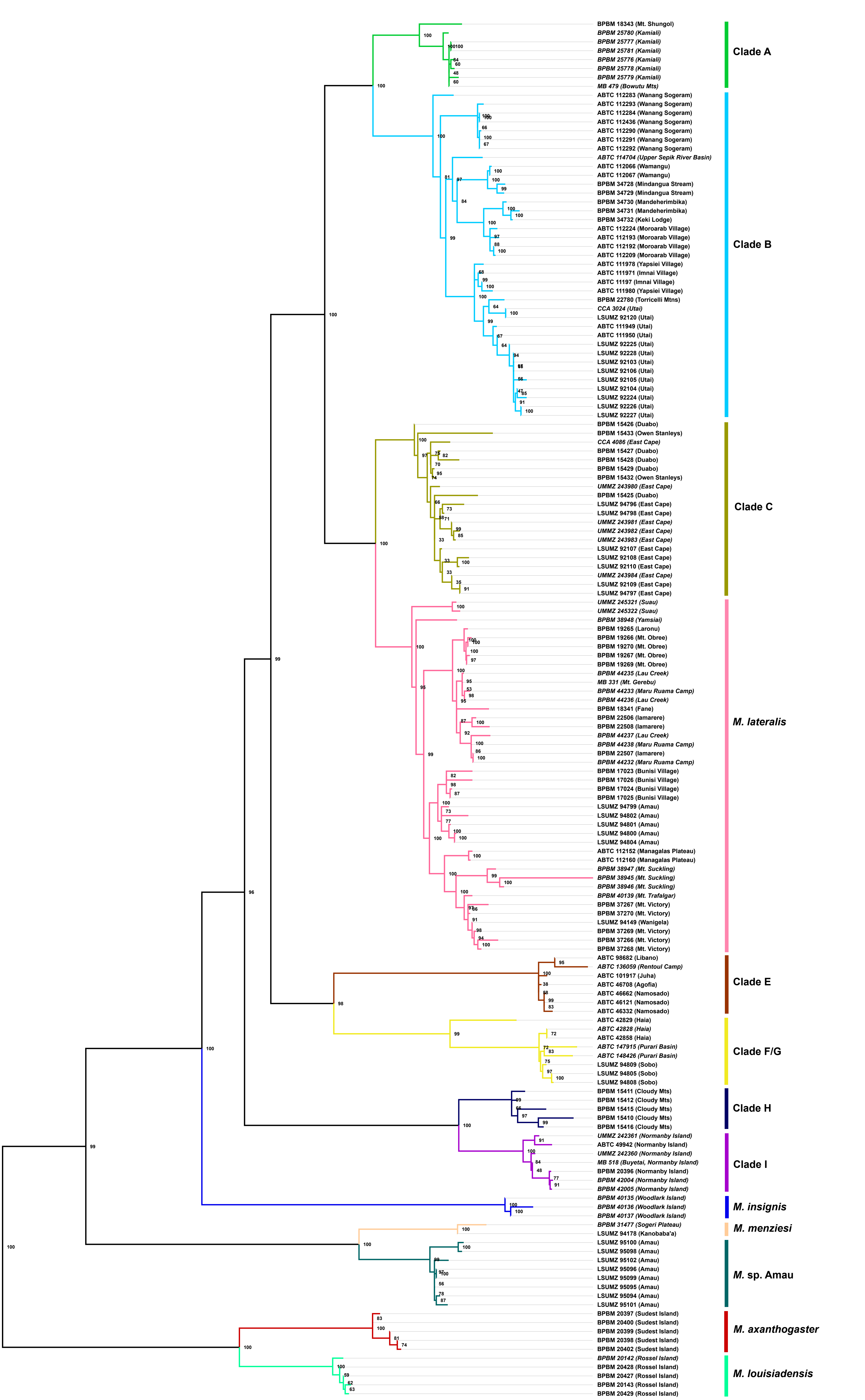

Supplement: Supplementary file 6 — Appendix S6: Full maximum‐likelihood (ML) phylogeny using concatenated mitochondrial loci generated on the IQTREE Web Server. Museum catalogue numbers and localities are given at each tip. Sequences from samples added to this study in addition to those used by Oliver et al. (2013) are indicated by italics. ML support values for each node are given above branches. Lineages were allocated based on cytb p‐values > 4% and followed the lettering of Oliver et al. (2013). After we interrogated the sequence data for specimen ABTC42829, previously identified by Oliver et al. (2013) as a unique lineage ( M. lateralis Clade F), we determined a labelling or sequence error must have occurred in 12 s and, using data only for 16 s and cytb, found this specimen to fall within Clade G; we therefore created Clade F/G to remain consistent with the lettering used by Oliver et al. (2013). [file ECE3-16-e73291-s001.pdf]

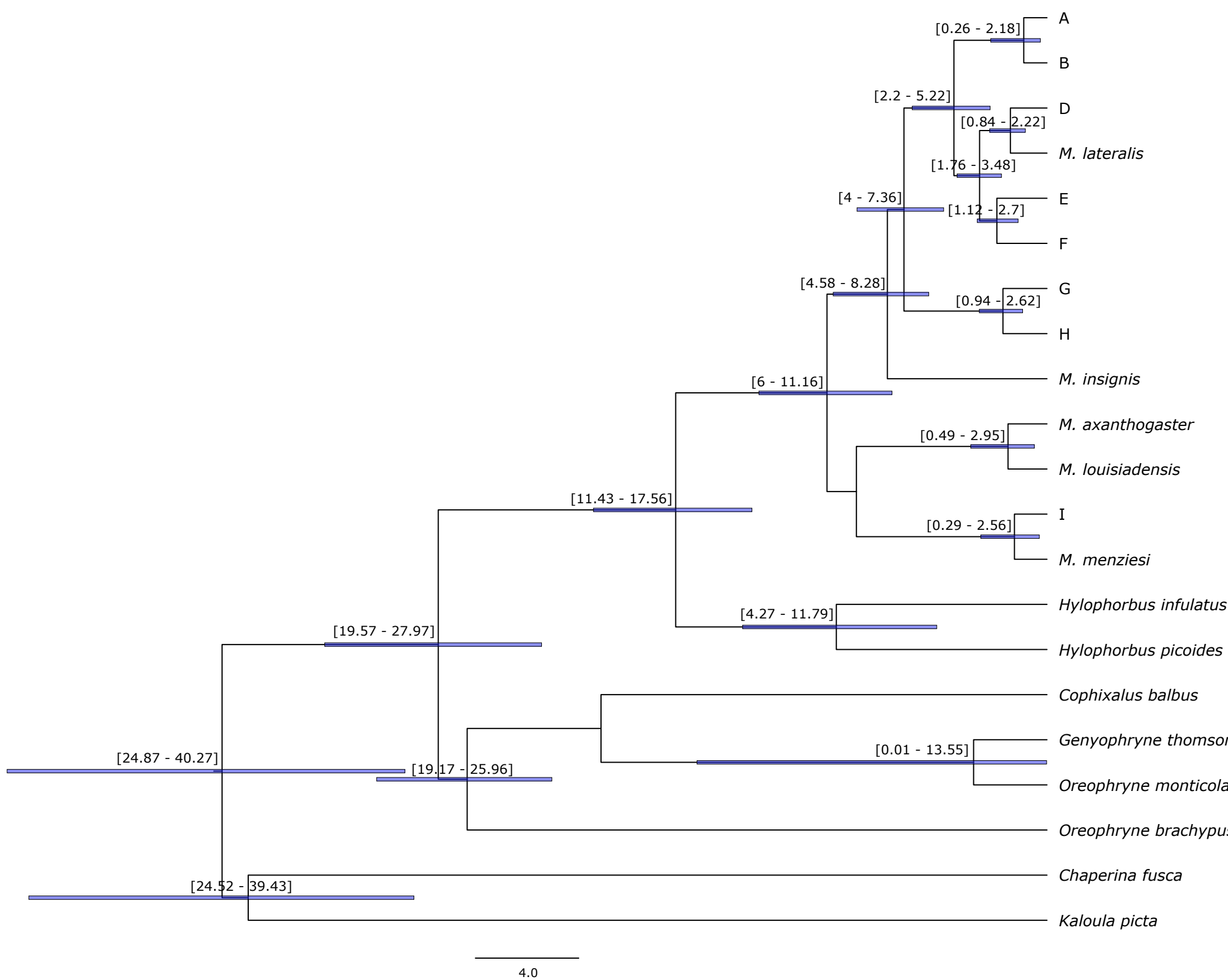

Supplement: Supplementary file 7 — Appendix S7: Time‐calibrated phylogeny of Mantophryne and outgroups. Scale bar indicates millions of years ago (MYA) and error bars at node are maximum and minimum estimates for divergence dates. [file ECE3-16-e73291-s005.pdf]
